# Dynamic Changes in Post-Ingestive Sensations after Consumption of a Breakfast Meal High in Protein or Carbohydrate

**DOI:** 10.3390/foods8090413

**Published:** 2019-09-14

**Authors:** Mette Duerlund, Barbara Vad Andersen, Derek Victor Byrne

**Affiliations:** Department of Food Science, Faculty of Science and Technology, Aarhus University, Kirstinebjergvej 10, DK-5792 Aarslev, Denmark; barbarav.andersen@food.au.dk (B.V.A.); derekv.byrne@food.au.dk (D.V.B.)

**Keywords:** post-ingestive sensation, appetite, satiety, consumer, protein, carbohydrate, breakfast

## Abstract

The obesity epidemic urges exploration of several parameters that play an important role in our eating behaviours. Post-ingestive sensations can provide a more comprehensive picture of the eating experience than mere satiety measurements. This study aimed to (1) quantify the dynamics of different post-ingestive sensations after food intake and (2) study the effect of protein and carbohydrate on hedonic and post-ingestive responses. Forty-eight participants (mean age 20.4) were served a breakfast meal high in protein (HighPRO) or high in carbohydrate (HighCHO) on two separate days using a randomised controlled crossover design. Post-ingestive sensations were measured every 30 min, for 3 h post intake using visual analogue scale (VAS). Results showed a significant main effect of time for all post-ingestive sensations. HighCHO induced higher hedonic responses compared to HighPRO, as well as higher ratings for post-ingestive sensations such as Satisfaction, Food joy, Overall wellbeing and Fullness. HighPRO, on the other hand, induced higher ratings for Sweet desire post intake. The development of sensations after a meal might be important for consumers’ following food choices and for extra calorie intake. More detailed knowledge in this area could elucidate aspects of overeating and obesity.

## 1. Introduction

Public health issues, like the obesity epidemic currently unfolding, demand action and require multiple approaches [1]. It urges us as researchers to pursue and explore several parameters that could play an important role in our eating behaviours. In particularly, assessing subjective appetite and its relationship to eating can provide important insights into our overall intake behaviours [2,3]. The concepts of satiation (cessation of a meal) and satiety (fullness over time) has been in great focus in recent decades [4,5]. This, among other things, involves the study of ‘The Satiety Cascade’ which conceptualizes as various signals (sensory, cognitive, post-ingestive, post-absorptive) that relate to eating and the concepts of satiation and satiety [6]. Several modifications over time of ‘The Satiety Cascade’ reveal a complex and dynamic interplay of numerous factors involved in the process of eating, including satiety and other post-ingestive effects [7,8]. 

Significant focus in the appetite research field has been on quantifying satiety as well as measuring the satiating effects of different foods, especially foods differing in macronutrient content. Researchers attempt to determine if specific diet manipulations might obtain benefits related to satiation and satiety [4,9,10,11]. Particularly research on proteins’ effect on satiety has gained much attention [4,9,11,12,13,14,15,16,17]. In a research study with healthy women, high-protein-low-carbohydrate Greek yoghurt led to increased fullness and delayed subsequent eating compared to low-protein-high-carbohydrate yoghurt (iso-caloric) [15]. Moreover, Anderson and Moore (2004) conclude that the protein content of a food is a strong determinant of short-term satiety [16], and Morell and Fiszman (2017) state that protein is the most effective food macronutrient providing satiating effects [11]. 

However, asking consumers only about satiation and satiety in research studies does not provide the full picture on post intake experiences nor about the foods effect on these post intake experiences. Studies have shown that other sensations prove important to our hedonic experiences of food [12,18,19,20,21] and can explain post-meal desires [22] potentially leading to additional calorie intake. For instance, Andersen et al. (2017) found that, besides the sensation of fullness, also energy and psychological wellbeing were important positive drivers of food satisfaction, and that nausea negatively influenced food satisfaction [18]. Thus, other sensations also contribute to the consumers’ eating experience, and by including additional sensations, we obtain a more comprehensive picture of what lies behind and beyond satiety. It enables us to study which sensations contribute positively or negatively to the food experience as well as enables us to study how certain product factors contribute to these different post-ingestive sensations. Post-ingestive experiences therefore offer new premises to predict consumers’ food choice and better understand our eating behaviours [10,23]. The contribution of different sensations to food choice demonstrates importance, and researchers within Sensory and Consumer Science subsequently move from one-sip evaluations into a bigger picture focusing on the full consumer experience, including post-ingestive experiences [10,12,18,23,24,25,26]. This extended information empowers a deeper understanding of the complex interplay between appetite sensations and food intake. It enables insights into the importance and implications of different sensations to food preference and eating behaviour.

Exploring different post-ingestive sensations within the spectrum of appetite and eating experiences, allows consumers to connect to their ability to introspect on a conscious level. Interoception defines as our ability to sense internal bodily states such as hunger, satiety and energy, but also other perceptions like pain, temperature, muscular and visceral sensations. Interoception focuses on our subjective evaluations as well as our self-awareness e.g., ‘how I feel’ [27,28,29]. Evaluating interoceptive states provides us, as researchers, with consumers’ current conscious awareness and gives an in-depth picture of the spectrum of appetite [2]. The perception of bodily signals might therefore constitute crucial factors in understanding and regulating our overall food intake [28,30]. 

Consequently, assessing appetite should not merely consist of hunger and satiety measurements, but could include several other parameters e.g., ‘feeling good’, energy, satisfaction, relaxation, heartbeat detection, food pleasure and other subjective evaluations [23,24,26,31,32,33]. For instance, Meiselman (2016) accentuates that wellbeing and quality of life can be central additions when measuring consumer perceptions of food [26]. Research within wellbeing points towards relevance in Consumer Science and several researchers also introduce the concept of wellbeing in relation to food [23,26,32,34,35]. Andersen and Hyldig (2015) found physical wellbeing to function as an important element in food satisfaction, and that physical wellbeing also includes the sense of an appropriate energy level after intake [24]. Food studies have used variables such as psychological wellbeing, desire for other foods, food satisfaction, nausea, fulfilment of expectation, reflux, physical wellbeing, pleasantness, mental hunger, alertness, energy level when evaluating post-ingestive experiences [12,18,19,20]. Møller (2015) states that there is a need for methods quantifying pleasure and satisfaction obtained from eating food which should go beyond the actual eating event and rely on different types of memory and interoceptive states after eating [31]. 

Exploring beyond satiety and focusing on post-ingestive sensations also allows us to contribute further on the knowledge we have on macronutrients’ effects. Foods macronutrient content influences the satiating power of a food, but does it also influence other post-ingestive sensations besides satiety? Protein’s and carbohydrate’s role in more elaborated aspects of post-ingestive experiences remain unclear and gives rise to measuring post-ingestive sensations and their dynamics in relation to macronutrient content. One study by Boelsma et al. (2010), sought to measure postprandial wellness after intake of two protein-carbohydrate meals. They found that a liquid high-protein breakfast induced higher levels of specific parameters of wellness, such as satisfaction and pleasantness, than did a liquid high-carbohydrate breakfast. Postprandial wellness was measured using the variables satisfaction, pleasantness, sleepiness, relaxation, mental alertness and physical energy [12]. Additional research in this area is needed to establish further relationships, for example protein and carbohydrate’s effect on post meal desires as well as post meal wellbeing. Also, the relevance for food intake behaviour remains to be established [12,23]. 

From a scientific point of view, this study clarifies protein and carbohydrate’s effect on specific post-ingestive sensations. From an industry perspective, it is relevant to know what increased protein or carbohydrate content entail in terms of post intake experiences, as this can determine whether consumers choose a product and consequently consume it repeatedly. The study contributes with new knowledge about the sensations perceived in the time after intake, namely, how these sensations develop over time, defined by *the dynamics* of the sensations. Post-ingestive sensations occur after food intake (per definition) and for this study, post-ingestive sensations are defined as *‘the subjective perceptions of the body after eating’* as described by Duerlund et al. (2019) [23]. 

The present study seeks to unravel and understand the extended eating experience with its related post-ingestive sensations. This research thus situates itself in the area of understanding a more elaborated consumer experience and contributes to the diverse and complex area of appetite research. The overall aims for this research study were thus to:

(1) Quantify the dynamics of different post-ingestive sensations after food intake
(2) Study the effect of protein and carbohydrate on hedonic and post-ingestive responses.

The study involved development of a questionnaire and conducting of a consumer study serving a breakfast meal differing in macronutrient content. The test meals consisted of yoghurt either high in protein content (HighPRO) or high in carbohydrate content (HighCHO). 

## 2. Materials and Methods 

### 2.1. Participants

Participants included Danish students recruited from Ollerup Sports Academy located on Fyn, Denmark, via voluntarily sign-up after personal contact to the Academy and subsequent advertisement via their intranet (*n*_total_ = 48). Inclusion criteria involved being above 18 years of age, being a breakfast eater, being a liker of yoghurt, and not suffer from any food allergies. Table 1 displays the characteristics from the 48 participants including standard deviation and range. Prior to the study, participants gave their written consent. Ethical approval is not required for this type of study according to the National Committee on Health Research Ethics in Denmark (Section 14 (2) in the Committee Act) [36]. For their participation, students received a cinema ticket for an optional movie at a cinema close by. 

### 2.2. Study Design and Procedure

**Figure 1:**
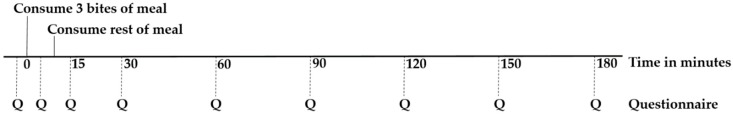
Schematic overview of the study with time intervals for answering of questionnaire.

The study was conducted as a central-location-test at the Ollerup Sports Academy utilizing a randomised controlled crossover design. Each participant came for two separate breakfast sessions on two separate consecutive days. The test meals were served in random order across the participants. On the test day, participants came fasting since 22:00 the night before and the study started in the morning at 7:30 a.m. for breakfast and ran for 3 h until 10:30 a.m. The breakfast meals were obligatory intake to make sure that all participants consumed the same amount of calories. Subjective measurements were collected pre intake, during intake (after three bites), and in 30 min intervals until three h post intake (T = 0, 15, 30, 60, 90, 120, 150, 180 min). See schematic overview of the study design in Figure 1.

### 2.3. Test Meals

The two test meals consisted of yoghurt with an added topping of plain muesli, almonds, raisins, and fresh blue berries, which is considered a typical breakfast in Denmark. The topping stayed consistent for the two meals, and the purpose was to create an appealing breakfast meal (visual, flavour, texture) rather than just yoghurt. The two meals were iso-caloric (393 Cal), but differed in protein and carbohydrate content, producing a high-protein test meal (HighPRO) and a high-carbohydrate test meal (HighCHO). The yoghurt was commercially bought (Arla^®^ lactose-free yoghurt natural) as the base and then added protein (whey protein isolate, Lacprodan^®^ SP-9225 Instant, Arla Foods, Viby, Denmark) or carbohydrate (glucose syrup, Dansukker^®^, Copenhagen, Denmark). The test meals were made following standardized procedures to ensure validity and standardization. For specification and content of the two test meals, see Table 2. 

### 2.4. Questionnaire and Post-Ingestive Response Variables

The questionnaire and chosen post-ingestive response variables were developed based on existing scientific literature as well as results from a previous qualitative focus group study conducted by the authors about consumer reflections on post-ingestive sensations [12,18,20,23]. Measurements were collected using visual analogue scale (VAS) via Compusense® Cloud software (Compusense Inc., Guelph, Ontario, Canada) in randomized order. Data was thus collected on a continuous scale ranging from 0, anchored “not at all” to 10, anchored “extremely”. The full questions were developed and phrased in the participants’ mother tongue, Danish. Appendix A displays the original Danish phrasing of the questions as well as the translated English phrasings. Additionally, all participants filled out an extra demographics questionnaire including gender, age, weight and height. The included response variables are presented in Table 3. Sensory satisfaction refereed to the hedonic experience of the meal’s sensory properties during intake, whereas Satisfaction refereed to a general positive response to the food after intake [19].

### 2.5. Data Analysis

Data on self-reported weight and height were used to calculate Body Mass Index (BMI): weight (kg)/(height (m))^2^. Analysis of variance (ANOVA) was used to test effect of breakfast meal on hedonic response variables. Repeated measures ANOVA was applied to analyse dynamics in the post-ingestive sensations over time for each test meal. We applied the option REstricted Maximum Likelihood (ReML) with an Autoregressive covariance structure, which assumes that correlation is the highest between consecutive measures and declines for measures further apart. Repeated measures ANOVA is appropriate when repeated scores are made sequentially within products and assumes non-independent measurements over time. Mauchly’s Sphericity test was applied to show significant non-independence and thereby justify the use of repeated measures ANOVA. Repeated measures ANOVA, option least squares (LS) was applied on centred data to analyse if the change in post-ingestive sensations over time was different for each test meal. *p*-values < 0.05 were considered statistically significant, and Fisher’s Least Significance Difference (LSD) test was applied to account for multiple comparisons. Effect sizes were examined using Cohen’s d values [37]. All statistical analyses of the data were carried out using XLSTAT by Addinsoft, version 2018.6. (XLSTAT, Long Island, NY, USA)

## 3. Results

### 3.1. Hedonic Differences between Test Meals

After consuming three bites of the test meal, participants evaluated three hedonic questions: Overall liking, Desire-to-eat the rest of the portion, and Sensory satisfaction. A difference was seen between the two test meals with significantly higher ratings for HighCHO compared to HighPRO for all hedonic response variables, Table 4. 

### 3.2. Dynamics of Post-Ingestive Sensations

A highly significant effect of time (*p* ≤ 0.0001) was seen for all post-ingestive sensation variables across test meals: Energized (F = 18.45, *p* < 0.0001), Relaxation (F = 5.38, *p* < 0.0001), Concentration (F = 13.89, *p* < 0.0001), Sleepiness (F = 7.42, *p* < 0.0001), Fullness (F = 88.78, *p* < 0.0001), Hunger (F = 64.22, *p* < 0.0001), Overall wellbeing (F = 12.65, *p* < 0.0001), Physical wellbeing (F = 9.58, *p* < 0.0001), Mental wellbeing (F = 6.95, *p* < 0.0001), Desire-to-eat (F = 53.18, *p* < 0.0001), Sweet desire (F = 4.42, *p* < 0.0001), Salty desire (F = 6.07, *p* < 0.0001), Fatty desire (F = 4.90, *p* < 0.0001), In need of food (F = 48.72, *p* < 0.0001), Food joy (F = 4.92, *p* < 0.0001) and Satisfaction (F = 5.05, *p* < 0.0001).

This shows that all the response variables change significantly over a period of three h after consumption of these breakfast meals. The 3-h (0–180 min) dynamics for post-ingestive sensations for each test meal, HighPRO and HighCHO respectively, are presented in Table 5 and Table 6.

Subjective measures for Energized significantly increased with large effect sizes (*p* < 0.0001, HighCHO: d = 0.9, HighPRO: d = 1.1) after consumption for both test meals and peaked with a maximum score (4.55) for HighPRO at T = 15, and a maximum score for HighCHO (4.79) at T = 60, where after the sensation of Energized decreased again. 

Subjective measures of Fullness significantly increased (*p* < 0.0001, HighCHO: d = 2.4, HighPRO: d = 2.1) immediately after consumption of both test meals (T = 15), where it reached its highest mean ratings as well (HighPRO: 5.53, HighCHO; 5.79). Thereafter Fullness sensation gradually decreased until T = 180, to the same as the pre intake baseline measurement at T = 0. So comparing T = 0 and T = 180, there was no significant difference in Fullness, suggesting that three h is the time point where Fullness sensation resets itself when consuming these meals in these amounts. 

Development over three h for Sleepiness started with the highest mean score at T = 0 for both test meals (HighPRO: 5.93, HighCHO: 6.34), and then significantly decreased (*p* < 0.0001, HighCHO: d = 0.6, HighPRO: d = 0.5) immediately after consumption (T = 15). Hereafter the rating of Sleepiness continuously decreased until T = 60 for the HighPRO meal, and until T = 90 for the HighCHO meal, where it significantly (HighCHO: *p* = 0.010, d = 0.4; HighPRO: *p* = 0.042, d = 0.3) started to increase again and peaked at T = 120 and T = 150, after which rating of sleepiness again started to decline until T = 180. 

Subjective measures of Overall wellbeing showed a significantly increase (*p* < 0.0001, HighCHO: d = 0.9, HighPRO: d = 0.5) immediately after consumption (T = 15) for both test meals, and stayed constant for 60 min after consumption, with a significantly decrease 90 min after consumption (HighCHO: *p* = 0.021, d = 0.3; HighPRO: *p* = 0.021, d = 0.2), and returning to similar ratings as pre intake when reaching T = 180. 

Subjective measures of Food joy showed a gradual decline in mean ratings for both test meals during the whole period after consumption, starting at their highest mean ratings at T = 15. At 90 min after consumption, measures of Food joy dropped significantly compared to T = 15 (HighCHO: *p* = 0.015, d = 0.2, HighPRO: *p* = 0.026, d = 0.2). Hereafter, ratings of Food joy continued to decrease, and for both test meals there was a significant overall decrease in Food joy from T = 15 until T = 180 (*p* < 0.0001, HighCHO: d = 0.7, HighPRO: d = 0.5). Food joy was not measured pre intake (T = 0), since it was a measure related to the consumed test meal. 

For HighCHO, ratings of Sweet desire significantly decreased immediately after consumption (*p* = 0.023, d = 0.2), as opposite to HighPRO. For HighPRO, sweet desire ratings immediately increased after consumption (*p* = 0.050, d = 0.2), where after it decreased again until T = 60. Ninety min after consumption, Sweet desire ratings increased significantly until T = 180 for HighPRO (*p* < 0.0001, d = 0.5), with the biggest increase between T = 150 and T = 180 after consumption (*p* = 0.010, d = 0.2). From T = 120 until T = 180, Sweet desire ratings significantly increased after consuming for HighCHO (*p* < 0.0001, d = 0.6). 

Subjective measures for In-need-of-food significantly decreased (*p* < 0.0001, HighCHO: d = 1.3, HighPRO: d = 1.4) immediately after consumption (T = 15) for both test meals. Hereafter, In-need-of-food showed a steep incline for the rest of the period until T = 180, where it reached its highest mean ratings with significantly higher ratings than pre intake (HighPRO: *p* = 0.022, d = 0.2, HighCHO: *p* < 0.0001, d = 0.8). Between T = 90 and T = 120, the dynamics of In-need-of-food ratings showed a crossover between the two test meals, with HighPRO changing from having higher In-need-of-food ratings to lower In-need-of-food ratings than HighCHO. 

Consumer’s rating of Satisfaction was the highest immediately after consumption (T = 15), and then decreased throughout the three h. This trend shows for both test meals, and for HighCHO, Satisfaction significantly decreased between T = 30 and T = 60 (*p* = 0.024, d = 0.2), and then again between T = 120 and T = 150 (*p* = 0.017, d = 0.2). 

### 3.3. Effect of Test Meal on Post-Ingestive Sensations

**Figure 2:**
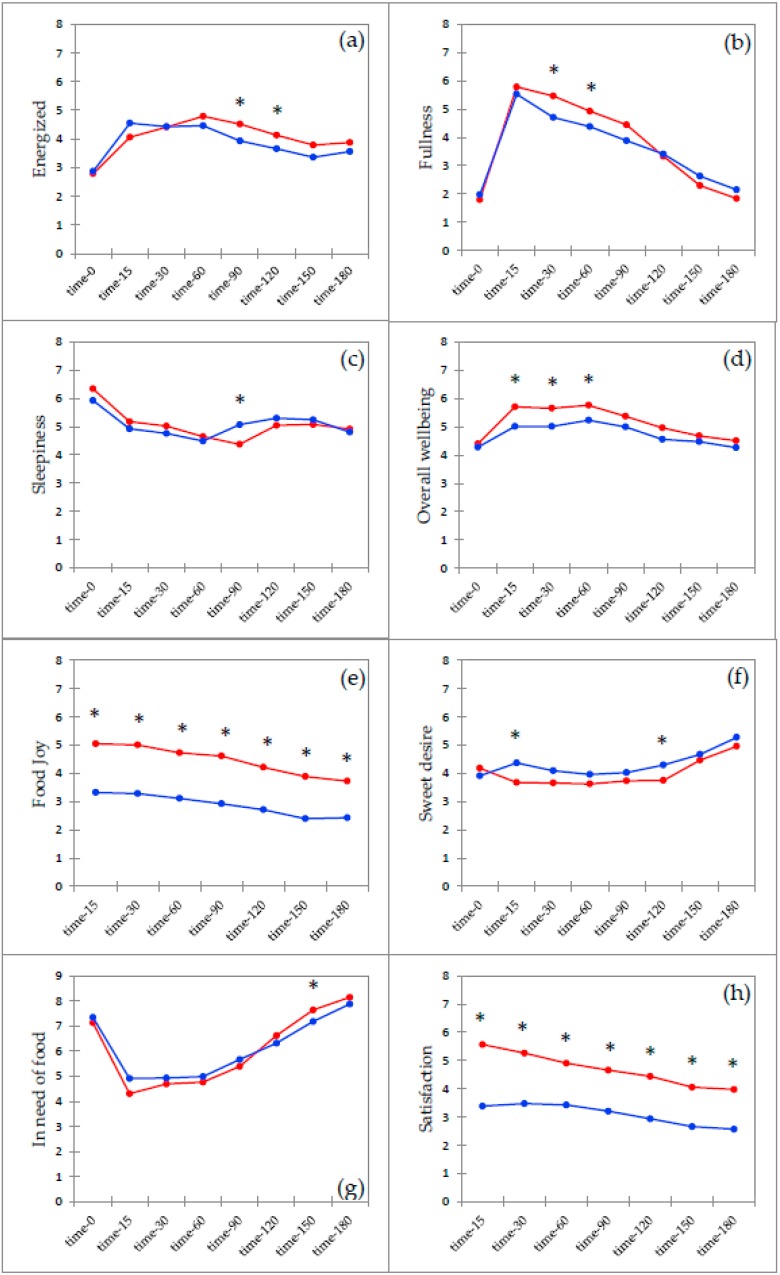
Post-ingestive responses at time-0 (pre intake),15,30,60,90,120,150,180 for Energized (**a**), Fullness (**b**), Sleepiness (**c**), Overall wellbeing (**d**), Food joy (**e**), Sweet desire (**f**), In need of food (**g**), Satisfaction (**h**). Red = High-carbohydrate test meal, Blue = High-protein test meal. Asterisk (*) indicates significant difference between the two test meals for the specific time point (*p* < 0.05).

For an overview of the significant differences (*p* < 0.005) between HighCHO and HighPRO see Figure 2, where an asterisk indicates significant differences at that time point. Only selected post-ingestive variables are displayed in the figure. Pre intake (T = 0), no difference for the two test meals were found for any of the post-ingestive variables. For the sensations of Satisfaction and Food joy, results showed significant differences between HighPRO and HighCHO for the whole period of the three-hour study (*p* < 0.0001, d = 0.8), with the HighCHO inducing significantly higher (*p* < 0.0001, d = 0.8) ratings than the HighPRO meal. Energized sensation showed a significantly higher rating for the HighCHO meal at 90 (*p* = 0.014, d = 0.4) and 120 (*p* = 0.049, d = 0.3) min after consumption, whereas Sleepiness ratings showed significantly higher ratings for HighPRO at T = 90 (*p* = 0.023, d = 0.4). Significantly Fullness differences between the two test meals were found at 30 (*p* = 0.013, d = 0.4) and 60 (*p* = 0.031, d = 0.3) min after consumption, with HighCHO depicting the highest Fullness sensations at these time points. When reaching T = 180 both test meals showed no differences in Fullness. Both hunger and In-need-of-food variables significantly differed between the two test meals 150 min (Hunger: *p* = 0.011, d = 0.4, In-need-of-food: *p* = 0.040, d = 0.3) after consumption with HighCHO inducing the highest ratings for both variables. Same trend was shown for Desire-to-eat-something, where HighCHO had significantly higher ratings than HighPRO at T = 150 (*p* = 0.029, d = 0.3). Significant differences between the two test meals were seen at T = 15 for Overall wellbeing (*p* = 0.009, d = 0.5), Physical wellbeing (*p* = 0.012, d = 0.4) and Mental wellbeing (*p* = 0.033, d = 0.3) with HighCHO rating the highest compared to HighPRO. Overall wellbeing showed significant meal differences for the first three time intervals (T = 0, 15, 30), and hereafter no differences were observed between HighCHO and HighPRO. Ratings for Sweet desire differed significantly between the two meals with HighPRO inducing higher Sweet desires at T = 15 (*p* = 0.035, d = 0.3) and again at T = 120 (*p* = 0.035, d = 0.2). For Relaxation, Fatty desire, and Salty desire, no significant test meal differences were observed at any of the time points.

## 4. Discussion

### 4.1. Implications of Hedonic Parameters

Consumers’ overall hedonic acceptances of the two test meals revealed a significantly lower acceptance (with large effect sizes [37,38]) of the HighPRO test meal compared to the HighCHO test meal for all three hedonic variables: Overall liking, Desire-to-eat the rest, and Sensory satisfaction. Increasing the protein content of a meal can significantly decrease its palatability [4,39]. A reason in this study might be an off-flavour of whey protein isolate that can carry through to the meal. It is well known that off-flavour of whey protein is a primary negative driver of acceptance [40]. Attributes that could have influenced the consumers’ ratings in this study are potential mouth-drying and chalky mouthfeels in the HighPRO meal compared to the sweet and mouth-coating attributes in the HighCHO meal [40,41]. However, these descriptions are not validated from a trained sensory panel. Additionally, sweetness often functions as a strong influencer of pleasantness and palatability with a powerful hedonic appeal [42,43], and thus the HighCHO meal could have induced higher hedonic acceptance due to the sweet glucose syrup added. Martini et al. (2018) found reduced palatability for high-protein pasta formulations using protein sources from egg white and soy isolate indicating that protein is likely to negatively affect sensory properties of pasta except for appearance [13]. Conversely, Boelsma el al, (2010) found no clear difference in enjoyment of two meals differing in protein content being high protein/low carbohydrate or low protein/high carbohydrate meals. Both test meals by Boelsma et al. (2010), however, were added glucose syrup, as opposite to our study, where only the HighCHO test meal was added glucose syrup [12]. Boelsma et al. (2010) succeeded in their intention to keep their two test meals similar in hedonic aspects [12]. For future test meal development involving glucose syrup and/or whey protein isolate, consideration around sensory properties and the effects on consumer’s hedonic appreciation should carefully be taken into account. Standardizing test meals for sensory properties and hedonic appeal then enables a clearer picture of product effects on evaluated parameters. 

### 4.2. Satisfaction and Food Joy

In general, we saw that the HighCHO test meal induced highest ratings for both Satisfaction and Food joy. For both Satisfaction and Food joy, the questions were phrased so that the consumers should have in mind the test meal they ate e.g., “How satisfied are you with the breakfast meal you ate today?”, and “Please rate your sense of joy when thinking of the meal you ate today”. The evaluation of these sensations hence related to the memory of the test meal. The results could imply that when consumers rate Satisfaction and Food joy, they generally associate and refer to their hedonic acceptance, here being overall liking and the Sensory satisfaction, which they experienced during consumption. As mentioned, HighCHO rated significantly higher compared to HighPRO for hedonic acceptance in this study. Mattes and Vickers (2018) researched whether food-liking influenced hunger and fullness. They suggest, from their studies, that if people eat a food they greatly enjoy, instead of eating a less-well-liked version, they will experience more pleasure, satisfaction, and satiety [44]. We did see that the well-liked HighCHO test meal, in addition to Satisfaction and Food joy, also induced higher ratings for satiety at time points 30 and 60 min after consumption. Boelsma et al. (2010) found an association between satiety and feelings of satisfaction, indicating that satiety sensations may be important factors influential in the sense of wellness after consumption of food in general [12]. In addition, Andersen and Hyldig (2015) highlight the importance of sensory properties for food satisfaction. They found that sensory satisfaction was highly influential and predictive for food satisfaction in a consumer study with chicken soups [19]. Cardello et al. (2000) moreover suggest that satisfaction is a more appropriate measure of consumers’ response to foods than liking [45]. Having said that, it must be emphasized that our results demonstrated the sensations of both Satisfaction and Food joy to last longer after consumption of the HighPRO test meal compared to the HighCHO test meal. For Satisfaction, we saw a significant time effect in decline after 120 min for the HighPRO test meal, whereas this significant decline occurred already after 60 min for the HighCHO test meal. This indicates that more than the hedonic evaluation is part of the consumers’ sense of satisfaction. This is also pointed out by research signifying that multiple factors contribute to food satisfaction including hedonic experience as well as other post-ingestive sensations [18,24,46] For Food joy, the significant decline occurred after 90 min with the HighPRO, but already after 60 min with the HighCHO. Nevertheless, the HighCHO test meal rated significantly higher overall for both Satisfaction and Food joy at all time points, so interpretation of these findings should be taken with care. More studies on dynamics and quantification of Food satisfaction, pleasure, and post-ingestive sensations from eating foods are encouraged [18,19,31]. 

### 4.3. Measuring Dynamics of Post-Ingestive Sensations

Consuming the test meals induced various dynamics of post-ingestive sensations over the three-hour period post intake. We saw a significant effect of time for all the evaluated post-ingestive variables, supporting the relevance and use of post-ingestive response variables as measures of post food experiences, as well as showing consumers’ ability to sense and report these sensations in general. Andersen et al. (2017) also showed significant time effects for thirst, fullness, and energy level, but not for hunger, physical wellbeing, or psychological wellbeing in a time period of 40 min post intake of four fruit drinks [18]. Measuring satiety and post-prandial wellness, Boelsma et al. (2010) found that differences between their test meals stood particularly evident at about 3–4 h after consumption, this with a high-protein/low-carbohydrate meal rating higher than a low-protein/high-carbohydrate liquid breakfast meal [12]. Our measurements ended after 3 h, but the results display rather the opposite, that especially for Fullness and for Overall wellbeing, the differences were primarily evident earlier, at 30 and 60 min after intake, with highest ratings induced by the HighCHO test meal, not the HighPRO test meal, although only with small to medium effect sizes as classified by Cohen (1988) [37]. We do not know the subsequent development of post-ingestive sensations after the 3-h study period for this study. However, we believe that these studies in general show that consumers can sense and express intensity differences in post-ingestive sensations beyond satiety, and that the intensity discrepancies between studies are caused by the different foods eaten rather than consumers’ ability to perceive the sensations.

#### 4.3.1. The Dynamic of Sweet Desire

The dynamics for Sweet desire revealed an immediate incline for the HighPRO test meal and an immediate decline for the HighCHO test meal, showing a significant meal difference instantly after intake. Since the HighPRO test meal probably tasted less sweet than the HighCHO test meal with the added glucose syrup, these results indicate a development of sweet desire after eating something not sweet. Oppositely, after consuming the sweeter HighCHO test meal, the desire for sweet seems to decrease after consumption of sweet foods in this study. An explanation for this difference in Sweet desire dynamics could involve the theory behind sensory specific satiety (SSS) and sensory specific desires (SSD). SSS describes the decline in pleasantness of a food eaten relative to a food not eaten [47,48,49], but also suggests that it can reflect a decrease in both food liking as well as in food wanting for the eaten food after consumption. The latter was suggested by Haverman et al. (2009) from a study consuming chocolate milk [50]. SSD can be described as an intrinsic motivation to eat and general desire for certain foods [51,52], for instance sweet, salty, fatty desires evaluated in this present study. In a study by Harington et al. (2016), they found that the desire for sweet was maintained for their whole study period of three h after eating two slices of bread, whereas participants’ desire for salty, fatty and savoury decreased [53]. Another research study, looking at the effect of adding cayenne pepper to a soup, found an increase in sweet desire after consumption of a spiced soup compared to a non-spiced soup, suggesting alteration in sensory specific desires with the addition of spices to food [22]. In addition, Harington et al. (2016) suggest that our desire for sweet foods is partially disconnected from our appetite, hypothesizing a ‘dessert mentality’ [53]. The present study showed an immediate increase for both Fullness and for Sweet desire after consumption of the HighPRO test meal, indicating that these two sensations can be present at the same time. Recent work from Duerlund et al. (2019) found qualitative evidence from focus group interviews, expressing that it is possible to be full and still, at the same time, desire something else [23]. Murray and Vickers (2009) report similar results that one may feel physically full but mentally hungry. They suggest an overlap of hunger and fullness sensations, stating that it is possible to be slightly hungry and slightly full at the same time [54]. In a study by Lowe and Butryn (2007) they distinguish between homeostatic hunger and hedonic hunger driven by either the need for nutrients or a need for pleasure, respectively. They found that satiation did not seem to have an effect on hedonic hunger compared to homeostatic hunger [21]. We believe that mental hunger [54] and hedonic hunger [21] can relate to desires for specific sensory stimuli. Our findings suggest that one can experience an increase in fullness and at the same time feel an increase in sweet desire when consuming a non-sweet meal, this in accordance with the theory of SSS for one food and the development of SSD for something else. 

#### 4.3.2. Energized and Sleepiness as Two Opposites?

The post-ingestive sensation variables Energized and Sleepiness each displayed different dynamic curves in the three-hour study period after intake. Exploring consumer views of post-ingestive sensations, Duerlund et al. (2019) proposed that post-ingestive energy includes a positive general energy sensation, and a more negative low energy sensation e.g., feeling sleepy. In the qualitative study, it was mentioned that consuming specific foods could lead to sensations of heaviness, sleepiness, and lack of ability to focus [23]. In the present study, we saw that both Energized and Concentration increased immediately after consumption of food (with large effect sizes), whereas Sleepiness decreased (with medium effect size). Could these results indicate that food-induced post-ingestive sensations like feeling Energized and Sleepy function as two opposites of one pole? This question statement, however, undeniably requires more research for affirmation, validation, and elaboration. In relevance to energy and sleepiness, research suggests a phenomenon called ‘post-lunch sleepiness’ or a ‘post-lunch dip’ in energy. This is an innate tendency for sleep during the early afternoon, often more pronounced after consuming a heavy meal and/or a high fat/carbohydrate meal [23,55,56,57,58]. According to Monk (2005), this ‘post-lunch dip’ can occur even without having lunch, or one being unaware of the time of day [57]. In the present study, consumers had breakfast in the early morning and the results demonstrated that for the first 60 min the opposite to what is known as the ‘post-lunch-dip’ occurred. Here we saw that Sleepiness decreased, and Energized increased. With that said, Sleepiness increased again after 1 h post intake, but with the protein-rich meal inducing the highest Sleepiness ratings compared to the high-carbohydrate meal, suggesting that the ‘post-lunch sleepiness’ phenomenon did not apply to the present study design and results. Interpreting these results, this could have connection to the time of day and participants’ metabolic state. The participants came in fasting early morning, hence energy deficient, suggesting that any energy addition logically should lead to more energy. 

### 4.4. Product Factors and Their Effects on Post-Ingestive Sensations

The largest product effects in this study were seen for variables Food joy, Satisfaction and Overall wellbeing - all holistic variables, where the HighCHO rated significantly higher than the HighPRO, possibly leading back to consumer’s hedonic responses to the two test meals as discussed. Proteins’ role in this holistic area of post-ingestive sensations is not well-researched. Boelsma et al. (2010) sought to measure postprandial wellness after intake of two protein-carbohydrate meals, and found that a liquid high-protein breakfast induced specific parameters of wellness such as satisfaction and pleasantness more than a liquid high-carbohydrate breakfast did. This is the opposite for our study, where the high-carbohydrate meal induced higher Satisfaction, Mental-, Physical-, and Overall wellbeing than the HighPRO test meal. This difference may be due to a liquid product rather than a more solid product and the appropriateness of this. Often, high protein content causes a more viscous product, which may be more appropriate than a thin product for liquid breakfasts. Together with Boelsma et al. (2010) we state that more research is needed to establish further relationships and that the relevance for food intake behaviour remains to be established [12]. 

On the contrary, protein’s role on appetite and especially fullness/satiety is a well-researched area, and, in summary, high-protein diets seem to provide a tool for appetite control. Ergo, meals higher in protein tend to increase satiety compared to meals lower in protein, at least in the short term [4,11,16]. Interestingly, we found that the HighCHO meal induced higher Fullness ratings for the first two h after consumption, where after we observed a crossover to HighPRO inducing the highest Fullness, however not with a significant effect. For Hunger, In-need-of-food and Desire-to-eat, we saw this crossover just after 90 min and only with a significant product effect at 150 min after consumption. These results indicate a subjective shift in appetite state somewhere between 90 and 120 min after consumption dependant on intake of protein or carbohydrate content in this study. The HighCHO test meal started with a higher Fullness rating but also ended with a higher Desire-to-eat rating compared to the HighPRO test meal. There is still much we do not know about protein-induced satiety. This present study contributes with additional knowledge to unravel the many aspects of protein-induced satiety. It also point towards the need for studying more individual satiety sensations in order to gain insights into quantitative or qualitative effects of different types of protein as well as other macronutrients. For instance, Karalus and Vickers (2016) suggest 41 items to study within satiety questionnaires [20]. Many factors appear to influence satiety including palatability and type of protein. It is still not clear the amount and/or the type of protein that is required to promote satiety, and long term data are still limited and inconclusive within this research area [4,11,14,59]. 

### 4.5. Measuring Food-Related Wellbeing—Towards a Holistic Approach

The present research study goes beyond mere satiety and hunger measurements and holds a more holistic approach to consumer food experiences including the aspect of wellbeing. Wellbeing is a growing area of interest in research, and especially food-related wellbeing has in recent years gained much focus [22,26,34,35]. There exists no one universal definition or way to measure wellbeing as the concept proves complex, holistic and multidimensional [34,60,61,62] and moreover differs cross-culturally [32,35]. King et al. (2015) have developed a method, The WellSense Profile^TM^, which addresses consumer wellbeing in association with food. The method consists of five dimensions that encompasses wellbeing i.e., emotional, physical, social, intellectual, and spiritual [34]. In this study, we included certain and selected aspects of wellbeing, for instance Mental-, Physical-, and Overall wellbeing as well as Satisfaction and Food joy, all subjective and holistic measurements as suggested by Duerlund et al. (2019) and inspired by existing research on post-ingestive sensations, food satisfaction, and food reward/pleasure [12,18,21,23,63]. We also argue that variables such as Energized and Relaxation also contribute to food-related wellbeing. To support this, Andersen and Hyldig (2015) found that included in physical wellbeing is the sense of an appropriate energy level after intake and that physical wellbeing functions as an important determinant in food satisfaction [24]. The findings from this study adds to the relevance, importance and applicability of evaluating subjective food related wellbeing in consumer research, especially post food intake. Continuing research on food-related wellbeing helps us gain more knowledge into this complex and multifaceted concept as well as to how to best measure food-related wellbeing. Merely asking consumers to rate their mental and physical wellbeing post intake may not provide the most accurate measures, and perhaps more implicit measures of food-related wellbeing rather than just asking are needed. Coming studies from the authors will expand on the findings of post-ingestive sensations in relation to food reward and food joy/pleasure.

### 4.6. Study Considerations, Future Perspectives and Implications

In this study, we observed different post-ingestive dynamics following two breakfast meals either high in protein or carbohydrate. The present study shows that consuming food not only affects appetite sensations such as hunger and satiety. It also affects other post-ingestive sensations like feeling Energized, Sleepiness, Desires and more holistic sensations such as overall wellbeing, Food joy and Satisfaction. The presented results can be considered a contribute to supporting the development of a general description of post-ingestive sensation dynamics. Yet, studies on broader food categories would provide more knowledge and data to support a general description of subjective post-ingestive sensation dynamics. 

Subjective measurements of post-ingestive sensations are not widely used within sensory and consumer science, and with this study, we contribute with new knowledge about the perceived sensations in the time after food intake. The present study showed that the post-ingestive sensations evolve differently over time and depending on product characteristics, in this case protein or carbohydrate. One thing is product factors and their effect on post-ingestive sensations as researched in this study. Another thing is post-ingestive sensations and their effect on food choice and eating behavior, for example post meal snacking providing additional calories. Consumers could learn from their own post-ingestive sensations and use them in their daily food decisions on a self-conscious level. Different post-ingestive sensations such as post meal desires or post meal wellbeing might influence future decisions on food and snack intake. For instance, different sensations might alter the specific food we choose to consume. Post-ingestive sensations could also modify the time intervals between meals or snacking or cause reduced or increased next intake. The contribution of the different sensations to food intake is therefore important, with the overall purpose to explore how post-ingestive sensations contribute to human eating behavior and food appreciation [12,18]. Further research on post-ingestive sensations’ influence on eating behavior is thus warranted.

The concept of post-ingestive sensations provides both research and industry with new opportunities to explore consumer experiences around eating, and in particularly this new area can facilitate development of new products designed to address and perhaps provoke/induce certain wanted/desired sensations to help regulate body weight, manage calorie intake, and perhaps avoid eating when not hungry. This approach focusing on consumers’ subjective post-ingestive sensations offers new promises to predict consumer food choices and can give further insight into (over)eating. Findings can therefore have impact in a broader context, such as implications for public health issues and obesity, which is one of our grand challenges in the world, evolving into a global epidemic [1,64]. The importance of post-ingestive sensations relative to prediction of longer-term food intake is so far unstudied. More and longer-term research is needed to support our findings and to gain more in-depth knowledge about post-ingestive sensations and their importance to consumer product acceptance and to their relevance in food choice and eating behavior. 

### 4.7. Limitations 

The present study showed that the post-ingestive sensations evolve differently over time depending on product characteristics, in this case protein or carbohydrate. A study limitation was, the varying sensory characteristics of the two test meals, inducing significantly higher liking and desire-to-eat for the HighCHO meal. The high palatability possibly affected the evaluation of other variables like Satisfaction and Food joy, as discussed under Section 4.1: *Implications of Hedonic Parameters.* Another aspect to consider includes the duration of the experiment. The study showed the development of post-ingestive sensations in the short term over a three h period. The effect of product factors and dynamics of post-ingestive sensations long term was not studied. Thus, we cannot exclude different effects long term. Furthermore, it is important to consider the nature and composition of the sample. The number of participants was *n* = 48 and all participants were young Danish students at a sports academy. In terms of this as a limitation, we consider it a representative number linked to precedent in the research area in previous papers, e.g., Martini et al. (2018), Boelsma et al. (2010) and Karalus and Vickers (2016) whom had 20, 21 and 30 participants respectively [12,13,20]. Our results however, whilst representative for active young consumers may vary when considered in relation to the general population. Thus, further confirmatory studies are required with differing population groups to determine the generalisability of the results found in the present study.

## 5. Conclusions

This quantitative research study contributed with knowledge on how different post-ingestive sensations develop after food intake. The study further elucidated differences between two breakfast meals either high in protein or carbohydrate. The work involved development of a questionnaire as well as conducting of a consumer study measuring product and time effects for subjective evaluations of post-ingestive sensations. The present study indicates that consuming foods not only affects satiety but also other post-ingestive sensations such as Energy, Desires, Food joy, Food-related Wellbeing and Satisfaction. Results showed a main effect of time for all measured post-ingestive response variables, validating that these sensations do evolve and change over a period of three h after food intake. The HighCHO induced higher hedonic responses compared to the HighPRO breakfast meal, as well as higher ratings for post-ingestive responses such as Satisfaction, Food joy, Overall wellbeing and Fullness. HighPRO, on the other hand, induced higher ratings for Sweet desire post intake. 

This research study indicates applicability of post-ingestive sensations in food studies that goes beyond mere satiety measurements. Post-ingestive sensations provide information important for consumers’ overall appreciation of products and including post-ingestive sensations and their dynamics when addressing the eating experience is therefore highly relevant. The development of sensations after a meal might be important for consumers’ following food choices and for extra calorie intake. Consumers could learn from their subjective sensations and this could influence their future decisions around eating. More detailed knowledge in this area might elucidate aspects of overeating and obesity. 

## Figures and Tables

**Table 1:** Participant characteristics.

Characteristics	
*n*_total_	48
Male/female	31/17
Age (years)	20.4 ± 1.09 (18–25) *
Weight (kg)	71.5 ± 11.99 (51–108) *
Height (cm)	175.7 ± 8.49 (162–192) *
BMI (kg/m^2^)	23.0 ± 2.41 (19–30.5) *

* Mean ± standard deviation (range). BMI—body mass index.

**Table 2:** Content of the two test meals: High-protein breakfast and High-carbohydrate breakfast.

Food Ingredient	High-Protein		High-Carbohydrate	
	Amount (g)	Calories (Cal)	Amount (g)	Calories (Cal)
Yoghurt ^1^	300	123	300	123
Lacprodan ^2^	35	131.5	-	-
Glucose syrup ^3^	-	-	42.35	131.5
Muesli	30	99	30	99
Almonds	6	30	6	30
Raisins	2.5	5	2.5	5
Fresh blueberries	2.5	5	2.5	5
Total	376	393	383.35	393

^1^ Arla® lactose-free yoghurt natural; ^2^ Lacprodan® SP-9225 Instant (whey protein isolate); ^3^ Dansukker® glucose syrup.

**Table 3:** Response variables included in the questionnaire pre-, during-, and post intake.

Pre Intake (Immediately before)	During Intake (after Three Bites)	Post Intake (0–15–30–60–90–120–150–180 min)
Energy Relaxation Concentration Sleepiness Fullness Hunger Overall wellbeing Physical wellbeing Mental wellbeing Desire-to-eat Sweet desire Salty desire Fatty desire In-need-of-food	Desire to eat the rest Overall liking Sensory satisfaction	Energy Relaxation Concentration Sleepiness Fullness Hunger Overall wellbeing Physical wellbeing Mental wellbeing Desire-to-eat Sweet desire Salty desire Fatty desire In-need-of-food Food joy Satisfaction

**Table 4:** Mean (*n* = 48) hedonic ratings ± standard deviations of Overall liking, Desire-to-eat the rest and Sensory satisfaction after three bites of the two test meals: High-protein (HighPRO) and High-carbohydrate (HighCHO).

Response Variable during Intake (after Three Bites)	HighPRO	HighCHO	Difference		
			F	*p*-Value	d *
Overall liking	3.496 ± 2.1 ^b^	5.906 ± 1.6 ^a^	145.5	<0.0001	1.3
Desire-to-eat the rest	5.004 ± 2.6 ^b^	6.963 ± 1.6 ^a^	80.4	<0.0001	0.9
Sensory satisfaction	4.144 ± 1.8 ^b^	5.908 ± 1.3 ^a^	83.8	<0.0001	1.1

* Effect size (Cohen’s d). Means with different superscript (^a,b^) within a row differ significantly.

**Table 5:** Time effects and means values (*n* = 48) of post-ingestive sensations for **HighPRO (A)** at different time points after consumption.

A: HighPRO	Pre Intake	15 min	30 min	60 min	90 min	120 min	150 min	180 min
Energized	2.87 ^a^	4.55 ^d^	4.43 ^d^	4.47 ^d^	3.94 ^c^	3.66 ^bc^	3.36 ^b^	3.56 ^bc^
Relaxation	6.10 ^c^	5.58 ^b^	5.34 ^ab^	5.41 ^b^	5.64 ^bc^	5.17 ^ab^	5.27 ^ab^	4.90 ^a^
Concentration	3.57 ^a^	4.65 ^c^	4.70 ^c^	4.63 ^c^	4.41 ^c^	4.02 ^b^	3.85 ^ab^	3.76 ^ab^
Sleepiness	5.93 ^c^	4.92 ^ab^	4.76 ^ab^	4.49 ^a^	5.07 ^b^	5.30 ^b^	5.24 ^b^	4.81 ^ab^
Satiety	1.97 ^a^	5.53 ^f^	4.71 ^e^	4.38 ^e^	3.89 ^d^	3.41 ^c^	2.63 ^b^	2.14 ^a^
Hunger	7.24 ^e^	4.58 ^a^	4.84 ^ab^	5.17 ^b^	5.62 ^c^	6.25 ^d^	7.09 ^e^	7.92 ^f^
Satisfaction ^1^	-	3.39 ^d^	3.48 ^d^	3.43 ^d^	3.21 ^cd^	2.94 ^bc^	2.66 ^ab^	2.57 ^a^
Overall wellbeing	4.28 ^a^	5.02 ^b^	5.01 ^b^	5.24 ^b^	5.00 ^b^	4.56 ^a^	4.47 ^a^	4.26 ^a^
Physical wellbeing	4.61 ^bcd^	4.92 ^de^	5.18 ^e^	5.27 ^e^	4.70 ^cd^	4.33 ^ab^	4.38 ^abc^	4.09 ^a^
Mental wellbeing	4.80 ^bc^	5.10 ^cd^	5.31 ^de^	5.55 ^e^	5.06 ^cd^	4.80 ^bc^	4.66 ^ab^	4.33 ^a^
Desire to eat	7.56 ^ef^	4.78 ^a^	5.03 ^a^	5.53 ^b^	5.99 ^c^	6.47 ^d^	7.17 ^e^	7.93 ^f^
Sweet desire	3.92 ^a^	4.37 ^ab^	4.10 ^a^	3.96 ^a^	4.03 ^a^	4.30 ^ab^	4.67 ^b^	5.27 ^c^
Salty desire	2.60 ^a^	2.88 ^ab^	2.81 ^ab^	2.61 ^a^	2.64 ^ab^	2.96 ^b^	3.31 ^c^	3.83 ^d^
Fatty desire	2.81 ^ab^	2.68 ^a^	2.70 ^a^	2.67 ^a^	2.80 ^ab^	2.86 ^ab^	3.10 ^b^	3.91 ^c^
In need of food	7.35 ^d^	4.91 ^a^	4.93 ^a^	4.99 ^a^	5.66 ^b^	6.32 ^c^	7.18 ^d^	7.88 ^e^
Food joy ^1^	-	3.32 ^d^	3.29 ^d^	3.11 ^cd^	2.93 ^bc^	2.71 ^ab^	2.40 ^a^	2.42 ^a^

Means with different superscript (^a,b,c,d,e,f^) within a row differ significantly. ^1^ Satisfaction and Food joy only measured after intake and not pre intake.

**Table 6:** Time effects and means values (*n* = 48) of post-ingestive sensations for **HighCHO (B)** at different time points after consumption.

B: HighCHO	Pre Intake	15 min	30 min	60 min	90 min	120 min	150 min	180 min
Energized	2.79 ^a^	4.06 ^bc^	4.42 ^cde^	4.79 ^e^	4.52 ^de^	4.14 ^bcd^	3.79 ^b^	3.88 ^b^
Relaxation	5.95 ^e^	5.82 ^de^	5.64 ^cde^	5.78 ^cde^	5.48 ^cd^	5.34 ^bc^	5.00 ^b^	4.41 ^a^
Concentration	3.50 ^a^	4.80 ^cd^	4.75 ^cd^	5.02 ^d^	4.72 ^cd^	4.61 ^c^	4.45 ^bc^	4.08 ^b^
Sleepiness	6.34 ^d^	5.18 ^c^	5.02 ^bc^	4.64 ^ab^	4.37 ^a^	5.05 ^bc^	5.08 ^bc^	4.91 ^bc^
Satiety	1.79 ^a^	5.79 ^f^	5.46 ^f^	4.93 ^e^	4.44 ^d^	3.33 ^c^	2.29 ^b^	1.83 ^ab^
Hunger	7.15 ^d^	4.14 ^a^	4.49 ^ab^	4.76 ^b^	5.64 ^c^	6.70 ^d^	7.74 ^e^	7.88 ^e^
Satisfaction ^1^	-	5.57 ^d^	5.26 ^d^	4.90 ^c^	4.66 ^bc^	4.44 ^b^	4.06 ^a^	3.98 ^a^
Overall wellbeing	4.40 ^a^	5.71 ^cd^	5.66 ^cd^	5.76 ^d^	5.37 ^c^	4.97 ^b^	4.68 ^ab^	4.51 ^a^
Physical wellbeing	4.45 ^a^	5.56 ^cd^	5.71 ^d^	5.49 ^cd^	5.32 ^c^	4.92 ^b^	4.43 ^a^	4.24 ^a^
Mental wellbeing	4.86 ^ab^	5.62 ^c^	5.81 ^c^	5.82 ^c^	5.58 ^c^	5.15 ^b^	4.81 ^ab^	4.55 ^a^
Desire to eat	7.28 ^d^	4.67 ^a^	4.93 ^a^	5.06 ^a^	5.80 ^b^	6.72 ^c^	7.72 ^d^	8.37 ^e^
Sweet desire	4.18 ^b^	3.68 ^a^	3.66 ^a^	3.63 ^a^	3.73 ^a^	3.75 ^a^	4.47 ^b^	4.96 ^c^
Salty desire	2.56 ^a^	3.02 ^b^	2.79 ^ab^	2.98 ^b^	2.97 ^b^	3.11 ^b^	3.54 ^c^	4.02 ^d^
Fatty desire	2.69 ^b^	2.33 ^a^	2.60 ^ab^	2.73 ^b^	2.67 ^b^	2.89 ^bc^	3.16 ^c^	3.57 ^d^
In need of food	7.14 ^d^	4.31 ^a^	4.69 ^a^	4.77 ^a^	5.39 ^b^	6.62 ^c^	7.64 ^de^	8.15 ^e^
Food joy ^1^	-	5.05 ^d^	5.01 ^d^	4.73 ^cd^	4.61 ^c^	4.21 ^b^	3.89 ^ab^	3.73 ^a^

Means with different superscript (^a,b,c,d,e,f^) within a row differ significantly. ^1^ Satisfaction and Food joy only measured after intake and not pre intake.

## Appendix A. Phrasing of Questions

**Table A1:** Response variables with their original Danish phrasing a well as the English translation.

Variable	Original Danish Phrasing	Translated English Phrasing
Energy	“Hvor energisk er du lige nu?”	“How energetic are you right now?”
Relaxation	“Hvor afslappet er du lige nu?”	“How relaxed are you right now?”
Concentration	“Hvor koncentreret er du lige nu?”	“How is your concentration right now?”
Sleepiness	“Hvor træt er du lige nu?”	“How sleepy are you right now?”
Fullness	“Hvor mæt er du lige nu?”	“How full are you right now?”
Hunger	“Hvor sulten er du lige nu?”	“How hungry are you right now?”
Overall wellbeing	“I hvor høj grad fornemmer du en generel velvære lige nu?”	“Please rate your overall wellbeing as you feel it right now”
Physical wellbeing	“Hvor fysisk veltilpas er du lige nu?”	“Please rate your physical wellbeing as you feel it right now”
Mental wellbeing	“Hvor mentalt veltilpas er du lige nu maden?”	“Please rate your mental wellbeing as you feel it right now”
Desire-to-eat	“Hvor stor er din lyst til noget at spise lige nu?”	“How much do you desire to eat something right now?”
Sweet desire	“I hvor høj grad har du lyst til noget sødt lige nu?”	“How much do you desire to eat something sweet right now?”
Salty desire	“I hvor høj grad har du lyst til noget salt lige nu?”	“How much do you desire to eat something salty right now?”
Fatty desire	“I hvor høj grad har du lyst til noget fedt lige nu?”	“How much do you desire to eat something fatty right now?”
In-need-of-food	“I hvor høj grad mangler du noget mad lige nu?”	“How much do you need food right now?”
Food joy	“I hvor høj grad fornemmer du en glæde ved den mad du har spist i dag?”	“Please rate your sense of joy when thinking of the meal you ate today”.
Satisfaction	“Hvor tilfreds er du med det måltid du har spist i dag?”	“How satisfied are you with the breakfast meal you ate today?”
Desire to eat the rest	“I hvor høj grad har du lyst til at spise resten af portionen?”	“How much do you desire to eat the rest of the serving?”
Overall liking	“Hvor godt kan du lide morgenmaden?”	“How much do you like the breakfast?”
Sensory satisfaction	“Hvis du tænker på morgenmadens udseende, lugt, smag og konsistens samlet set hvor tilfredsstillet er du så?”	“If you consider the breakfast’s appearance, smell, taste, and consistence as a whole, how satisfied are you?”
